# Evaluation of Family Caregivers’ Use of Their Adult Care Recipient’s Patient Portal From the 2019 Health Information National Trends Survey: Secondary Analysis

**DOI:** 10.2196/29074

**Published:** 2021-10-04

**Authors:** Minakshi Raj, Bradley Iott

**Affiliations:** 1 Department of Kinesiology and Community Health University of Illinois at Urbana Champaign Champaign, IL United States; 2 Department of Health Management and Policy University of Michigan School of Public Health Ann Arbor, MI United States

**Keywords:** informal caregivers, family caregivers, patient portal, electronic health record, telehealth, aging in place, web-based medical record

## Abstract

**Background:**

Patient engagement is critical for realizing the value of telehealth modalities such as the patient portal. Family caregiver engagement may also be critical for facilitating the use of the patient portal among adult patients, including older adults.

**Objective:**

This study aims to analyze the 2019 Health Information National Trends Survey to characterize family caregivers’ use of their care recipient’s patient portal in terms of sociodemographic, health, and caregiving characteristics and caregivers’ use of their own portal.

**Methods:**

We conducted a secondary analysis of cross-sectional data from the National Cancer Institute’s Health Information National Trends Survey 5 Cycle 3. This survey was administered to 5438 US adults between January and May 2019. We analyzed data from 320 respondents who were identified as family caregivers. We created measures to reflect family caregivers’ use of their care recipient’s and their own portal, caregiver demographic and caregiving characteristics, and care recipient health characteristics.

**Results:**

Over half of the caregivers (179/320, 55.9%) reported using their own portal at least once, whereas only one-third (105/320, 32.8%) reported using their care recipient’s record in the previous 12 months. Caregivers using their own portal were significantly more likely to use their care recipient’s portal (odds ratio 11.18; *P*<.001).

**Conclusions:**

Policies should enable patients to designate family caregivers who can access their patient portal. Providers could screen caregivers for challenges in their caregiving responsibilities that may be addressed through the portal so they can better support their adult relatives. Interventions to support family caregivers, especially older caregivers, in using their own portal may facilitate their use of their care recipient’s portal.

## Introduction

### Background

Telehealth (ie, synchronous or asynchronous distribution of health services and information via electronic information or telecommunication) can fill gaps in health care delivery, including increasing access to services, reducing patient and family burdens (eg, transportation), and alleviating the impacts of provider shortages [1]. This is particularly the case among older adults who may experience greater barriers to access because of transportation and mobility challenges, with implications for continuity of care and ability to age in place [2]. However, there is a need to build evidence of patient engagement with telehealth modalities, such as the patient portal, to contribute to its value in care delivery for patients and their family caregivers [1].

Family caregivers are increasingly involved in health care and medical responsibilities, including communicating with clinicians, supporting medical decision-making, and assisting adult care recipients with following clinician recommendations [3,4]. Despite being responsible for communicating with their care recipient’s health care providers, few family caregivers use their care recipient’s patient portal [5]. Studies report that about 40% to 50% of patients use their patient portal [6,7] and that only 25% to 35% of caregivers, including family members and nonrelatives, use the portal for their caregiving responsibilities [5,8]. Two of the major challenges caregivers report include unmet information needs regarding their care recipient’s health conditions and difficulties in communicating with their care recipient’s provider; these challenges can have implications for caregiver distress and burden [9]. Providing caregivers with access to their care recipient’s patient portal can support caregivers in their responsibilities of communicating with their care recipient’s clinicians and retrieving important information about their care recipient’s health care needs, with the potential for enabling effective patient care [9,10]. In particular, for those supporting adults, family caregivers’ inclusion in the patient portal can support aging in place or their relative’s ability to live in their own home or community independently by facilitating remote communication and ensuring provision of clinical advice, medication refills, and viewing of laboratory results and referrals without making the care recipient susceptible to potential barriers such as transportation or mobility [11,12].

Previous studies characterizing the use of the patient portal suggest that low engagement with the patient portal for certain populations, such as older adults, could be attributed to technology barriers or to the possibility that family caregivers are using the patient portal on their behalf [6,7]. Although some caregivers have formal proxy access to their care recipient’s portal (with estimates at less than 5%), some literature suggests that 25% to 50% of proxies informally use their care recipient’s portal [13-15]. By offering caregivers log-in credentials for informal proxy access, patients may share more health information than intended or desired [16]. Furthermore, only two-thirds of surveyed American hospitals were found to provide the option of granting proxy access, and the process of doing so was often time consuming and challenging [17]. In addition, proxy access tools may not provide patients with the flexibility to choose which information should be shared with caregivers [15,18]. In general, studies suggest low use of the patient portal among family caregivers despite the potential benefits for adult care recipients, including aging in place, early detection of health needs, and continuity of care [2,5,16,19,20]. Prior work has demonstrated opportunities for patient portal use to aid family caregivers, including formally designating caregivers on patients’ medical records, supporting caregivers in their caregiving roles via training and access to behavioral health services, supporting caregivers in health care tasks, and expanding portal functionality to address caregivers’ unique needs [21]. Further, little research has examined how caregivers’ use of their own patient portal is related to their use of their care recipient’s patient portal [22]. Family caregivers who use their own patient portal may be more likely to use their care recipient’s patient portal, whereas those who do not use their own patient portal may have concerns about using the technology or may have concerns about using their own patient portal that translates to similar concerns about using their care recipient’s portal. Caregivers might also be more likely to access their care recipient’s patient portal if they anticipate a specific need, for instance, to obtain important laboratory test results or communicate with a provider about an upcoming surgery [14].

### Objectives

Unless family caregivers are effectively integrated into telehealth modalities such as the patient portal, there is potential to exacerbate disparities in access to telehealth and subsequent impacts on health, for example, among older adults, adults with limited computer or internet access, and racial and ethnic minority adults who may face sociocultural barriers to effective care independently via telehealth [23]. Family caregivers may be critical for ensuring effective and equitable health care delivery via telehealth modalities, including the patient portal. The purpose of this study is to characterize family caregivers’ use of their care recipient’s patient portal in terms of caregivers’ use of their own patient portal, to better inform the development of policies and design technologies that support family caregivers in managing their own health and the health and health care of their care recipient.

## Methods

### Data Source

We used data from the National Cancer Institute’s publicly available Health Information National Trends Survey (HINTS). The nationally representative survey routinely collects information about the American public’s use of cancer-related information and seeks to understand how adults aged ≥18 years use different types of health communication to obtain health information for themselves and for their relatives. We performed a secondary analysis of data from the HINTS 5 Cycle 3, administered to 5438 US adults between January and May 2019 [24].

### Study Sample

We restricted our sample to respondents who were family caregivers. First, we excluded observations (n=197) with missing responses to the question: Are you currently caring for or making health care decisions for someone with a medical, behavioral, disability, or other condition? We excluded noncaregivers (n=4413); respondents who provided professional support or had missing information about whether they provided professional caregiving support (n=122); caregivers of multiple adult care recipients and sandwich generation caregivers (ie, supporting both a child or children and at least one adult care recipient; n=10); and caregivers of friends or nonrelatives (n=18) to focus on family members specifically. We also excluded caregivers of children (n=221) as the policy landscape for caregivers of children (often parents or guardians) is different from the policies governing caregiver access to the medical records of adult relatives. Finally, we excluded observations with missing data in any of our measures of interest described below (n=137), resulting in our final analytic sample of (N=320) family caregivers.

### Measures

Our outcome of interest was caregiver use of their care recipient’s medical record, based on the question: How many times did you access your care recipient’s web-based medical record in the last 12 months? Respondents indicated the number of times they accessed the record from options: none, 1-2 times, 3-5 times, 6-9 times, and 10 or more times. From this, we created an indicator variable reflecting “No use (0)” or “Use (1).”

We assessed caregiver use of their own patient portal based on the question: How many times did you access your web-based medical record in the last 12 months? Possible responses included: 0, 1-2 times, 3-5 times, 6-9 times, and 10 or more times. From this, we created an indicator variable reflecting “No use (0)” or “Use (1),” which reflected using the portal more than once. Other covariates included caregiver demographic characteristics (age in years, self-reported sex [male or female], race and ethnicity [White, non-Hispanic; Black, non-Hispanic; Hispanic; and other, non-Hispanic], education [high school or less, some college, and college graduate], and United States Department of Agriculture metropolitan indicator [nonmetro vs metro]), caregiver physical health conditions (diagnoses of cancer, diabetes, heart condition, lung disease, or multiple conditions), caregiver mental health condition (diagnosis of depression), caregiving experiences (relationship to care recipient [spouse or partner, adult child, and other relative]), hours spent caregiving per week (less than 20 and 20 or more), and care recipient’s health conditions (physical, cognitive, or both).

To better understand the variation in caregivers’ use of their own medical record, which may give us insight into caregivers’ familiarity with different uses of the portal and their subsequent engagement with their care recipient’s portal, we evaluated caregivers’ reported uses of their patient portals and reasons for not using their records. Measures indicating reasons for using the record included medication refills, correcting inaccurate information in the record, securely messaging health care providers or staff, adding health information such as side effects to share with provider, downloading health information to a personal device such as a phone, and using information to make a decision about treatment. Measures indicating reasons for not using the record included preference to speak with the provider directly, not having a way to access the website, concerns about the privacy or security of the website, difficulties logging in, discomfort or insufficient experience with computers, having multiple portals, and not having a need to use the patient portal.

### Statistical Analysis

We summarized descriptive statistics to characterize the sample and then conducted a multivariable logistic regression to estimate the relationship between our outcome of interest (caregiver use [any vs none] of the care recipient’s patient portal) and covariates (caregiver use of own patient portal, demographic, health, and caregiving characteristics). Finally, we summarized the uses of the portal among caregivers who reported accessing their portal and reasons for not using the portal, among those who reported not accessing their patient portal.

## Results

### Overview

The final sample included 320 family caregivers. The average age of respondents was 57.8 years (SD 13.6), with nearly half of the respondents aged ≥60 years (Table 1). Our sample was predominantly female (190/320, 59.4%) and White, non-Hispanic (206/320, 64.4%). Nearly half of our sample reported having high blood pressure or hypertension (142/320, 44.4%), and over one-quarter (83/320, 25.9%) reported having depression. Over half of our sample (165/320, 51.6%) provided care to a parent, and almost one-third (97/320, 30.3%) support a spouse or partner. All respondents supported their care recipient with a physical condition (eg, diabetes, cancer, and aging-related conditions) and 57.5% (184/320) supported their care recipient with a cognitive condition (eg, dementia) with over one-third (112/320, 35%) of the respondents reporting spending 20 or more hours per week toward caregiving.

**Table 1:** Family caregiver demographic and health characteristics and caregiving experiences (N=320).

Demographic characteristics			Values
**Age (years)**			
	Value, mean (SD)		57.8 (13.6)
	**Age group, n (%)**		
		18-59	164 (51.3)
		≥60	156 (48.8)
**Sex, n (%)**			
	Male		130 (40.6)
	Female		190 (59.4)
**Race and ethnicity, n (%)**			
	White, not Hispanic		206 (64.4)
	Black, not Hispanic		37 (11.6)
	Hispanic		46 (14.4)
	Other, not Hispanic		31 (9.7)
**Education, n (%)**			
	High school or less		48 (15)
	Some college		86 (26.9)
	College graduate		186 (58.1)
**USDA^a^ rural-urban classification, n (%)**			
	Nonmetro		29 (9.1)
	Metro		291 (90.9)
**Income, n (%; US $)**			
	Below 50,000		102 (31.9)
	50,000-99,000		108 (33.8)
	100,000 or higher		110 (34.4)
**Health conditions, n (%)**			
	Cancer diagnosis		50 (15.6)
	Depression diagnosis		83 (25.9)
	Diabetes diagnosis		63 (19.7)
	Heart condition diagnosis		27 (8.4)
	High blood pressure or hypertension diagnosis		142 (44.4)
	Lung disease diagnosis		39 (12.2)
**Caregiving experiences, n (%)**			
	**Relationship with the care recipient**		
		Spouse or partner	97 (30.3)
		Adult child	165 (51.6)
		Other relative	58 (18.1)
	**Hours per week spent caregiving**		
		<20 hours	208 (65)
		≥20 hours	112 (35)
	**Care recipient’s conditions**		
		Physical^b^	320 (100)
		Cognitive	184 (57.5)
Use of own patient portal, n (%)			179 (55.9)
Use of care recipient’s patient portal, n (%)			105 (32.8)

^a^USDA: United States Department of Agriculture.

^b^Physical condition may include having any of the following conditions: cancer, orthopedic, musculoskeletal, another chronic condition (eg, diabetes), an acute condition, aging, or other**.**

Over half of respondents (179/320, 55.9%) reported using their own patient portal at least once, whereas 32.8% (105/320) reported using their care recipient’s portal at least once. We summarize respondents’ device ownership and engagement with different health technologies in Multimedia Appendix 1.

### Caregiver Use of Care Recipient’s Patient Portal

Family caregivers who use their own patient portal were significantly more likely to use their care recipient’s portal (odds ratio [OR] 11.18, 95% CI 5.51-22.69; *P*<.001; Table 2). Being female (OR 2.58, 95% CI 1.40-4.75; *P*=.002) was associated with a significantly higher likelihood of using their care recipient’s portal, whereas older caregivers (aged ≥60 years) were less likely to use their care recipient’s portal (OR 0.55, 95% CI 0.30-1.00; *P*=.05) and not having a diagnosis of depression (OR 2.24, 95% CI 1.13-4.46; *P*=.02) was associated with a significantly higher likelihood of using their care recipient’s portal. In addition, caregivers supporting a parent (OR 0.31, 95% CI 0.15-0.64; *P*=.002) and caregivers supporting another relative (OR 0.31, 95% CI 0.12-0.78; *P=*.01) were significantly less likely to use their care recipient’s portal when compared with caregivers supporting a spouse or partner.

**Table 2:** Odds ratios for family caregivers’ use of their care recipient’s patient portal in the last 12 months (N=320).

Characteristics			Odds ratio (SE; 95% CI)	*P* value
Caregiver use of own web-based medical record			11.18 (4.04; 5.51–22.69)	<.001
**Demographic characteristics**				
	**Age (years)**			
		18-59	Reference	N/A^a^
		≥60	0.55 (0.17; 0.30–1.00)	.05
	**Sex**			
		Male	Reference	N/A
		Female	2.58 (0.80; 1.40–4.75)	.002
	**Race and ethnicity**			
		White, not Hispanic	Reference	N/A
		Black, not Hispanic	0.60 (0.28; 0.24–1.52)	.28
		Hispanic	0.86 (0.37; 0.37–1.99)	.73
		Other, not Hispanic	0.53 (0.28; 0.19–1.47)	.22
	**Education**			
		High school or less	Reference	N/A
		Some college	0.53 (0.27; 0.20–1.44)	.21
		College graduate	0.88 (0.40; 0.36–2.13)	.78
	**USDA^b^ rural-urban classification**			
		Nonmetro	Reference	N/A
		Metro	0.89 (0.50; 0.30–2.68)	.83
**Health conditions**				
	**Cancer diagnosis**			
		Yes	Reference	N/A
		No	1.13 (0.46; 0.51–2.51)	.76
	**Depression**			
		Yes	Reference	N/A
		No	2.24 (0.79; 1.13–4.46)	.02
	**Diabetes**			
		Yes	Reference	N/A
		No	1.06 (0.39; 0.52–2.18)	.87
	**Heart condition**			
		Yes	Reference	N/A
		No	0.86 (0.45; 0.31–2.42)	.78
	**Lung disease**			
		Yes	Reference	N/A
		No	0.85 (0.36; 0.37–1.95)	.70
	**High blood pressure**			
		Yes	Reference	N/A
		No	0.84 (0.26; 0.46–1.53)	.57
**Caregiving experiences**				
	**Relationship to care recipient**			
		Spouse or partner	Reference	N/A
		Adult child	0.31 (0.11; 0.15–0.64)	.002
		Other relative	0.31 (0.15; 0.12–0.78)	.01
	**Hours per week spent caregiving**			
		<20 hours	Reference	N/A
		≥20 hours	0.97 (0.32; 0.51–1.86)	.94
	**Care recipient’s conditions**			
		Cognitive	1.05 (0.32; 0.58–1.89)	.88

^a^N/A: not applicable (for reference groups).

^b^USDA: United States Department of Agriculture.

### Caregivers’ Reported Reasons for Using or Not Using the Patient Portal

Caregivers who reported using the portal (n=179) most commonly reported using their own portal to look up test results (148/179, 82.7%), securely message their health care provider and staff (108/179, 60.3%), and request medication refills (96/179, 53.6%; Table 3). Among those who reported never using their own portal (n=141), the most common reasons included preferring to speak directly with their provider (99/141, 70.2%), not having a need to use the portal (76/141, 53.9%), not having a patient portal (38/141, 26.9%), and privacy and security concerns (36/141, 25.5%; Table 3).

**Table 3:** Caregivers’ reported uses of their own patient portal (n=179) and reasons for not accessing their own patient portal (n=141)^a^.

		Values, n (%)
**Caregivers’ reported uses of their own patient portal (n=179)**		
	Request correction of inaccurate information	13 (7.3)
	Download health information to computer or device	52 (29.1)
	Add health information to share with provider	58 (32.4)
	Help make decisions about treatment	70 (39.1)
	Request medication refill	96 (53.6)
	Securely message health care provider and staff	108 (60.3)
	Look up test results	148 (82.7)
**Caregivers’ reported reasons for not accessing their own web-based medical record (n=141)**		
	Have multiple web-based medical records	18 (12.8)
	Not comfortable or experienced with computers	25 (17.7)
	Do not have a way to access the website	27 (19.2)
	Difficulties logging into website	34 (24.1)
	Privacy and security concerns	36 (25.5)
	Do not have a web-based medical record	38 (26.9)
	Did not have a need to use web-based medical record	76 (53.9)
	Prefer to speak directly with provider	99 (70.2)

^a^Participants could indicate multiple reasons.

## Discussion

### Principal Findings

About one-third of the family caregivers in this study used their care recipient’s patient portal. Compared with family caregivers who do not use their patient portal, those who do are significantly more likely to use their care recipient’s portal. Consistent with previous findings, this study also suggests that family caregivers of a parent or another relative are significantly less likely to use their care recipient’s portal compared with caregivers of a spouse or partner [5,19]. Female caregivers are more likely to use their relative’s portal is less surprising, given that females are more likely to be caregivers. However, future research should study whether there are different uses of the patient portal across gender identities and the impacts of those uses on distress and ability to carry out caregiving responsibilities. However, older caregivers are less likely to use their care recipient’s patient portal compared with caregivers aged <60 years. Caregivers who access their portal reports include looking up laboratory results and communicating with their provider, whereas those who do not access the portal report reasons such as concerns about their preference to speak directly with their provider or not having a need to use the portal.

### Comparison With Prior Work

Our study aligns with previous research, suggesting that family caregivers may be using their care recipient’s patient portal on behalf of or with their care recipient [6,7]. However, engaging family caregivers in using their own portal may be critical for increasing the likelihood that they will use their care recipient’s portal either with or on behalf of their care recipient. This may be especially the case for older caregivers who may hesitate to use their own portal and be more likely to be a spouse or partner of their care recipient. Higher caregiver intensity (in terms of hours spent caregiving) is associated with a slightly lower likelihood of using their care recipient’s portal may be reflective of the burden associated with caregiving and its impact on caregivers’ time available to engage with the patient portal [25]. However, engaging with the patient portal could also reduce caregiver distress and burden, potentially alleviating challenges with communication and unmet information needs. This may be the case even among caregivers who do not perceive the need to use the portal. Achieving value in telehealth, particularly in terms of patient engagement, may require attention to family caregiver engagement [1,10].

Addressing family caregiver engagement with telehealth modalities will require the development of formal and standardized policies supporting family caregivers’ access to their care recipient’s patient portal [26]. Policies need to be developed to designate family caregivers who can use a patient’s medical record and determine the circumstances under which they can use it [27]. Policies also need to specify when patients should express their preferences for family caregiver designation (eg, upon enrollment with a physician) and how they can change their preferences or customize the types of information that are shown to family caregivers.

As evidence supports the role of family caregivers in promoting positive patient health outcomes, policies can also incentivize providers to support family caregivers in their health care responsibilities in various ways [28,29]. For instance, this could include training family caregivers on effective use of the patient portal to facilitate timely communication with providers about patient needs and concerns, refill medications, and fix inaccuracies in the record, among other possibilities. There may be opportunities to develop shared portal platforms for family caregivers and their care recipients, which could also offer resources and support for family caregivers. For instance, a shared portal could screen family caregivers for distress when they access their care recipient’s portal. Policy makers and payers must recognize family caregivers as integral components of value in telehealth. Subsequently, health care providers can encourage patients and their caregivers (or caregivers during their own health care visits) to use the patient portal as an approach to supporting caregivers so that they can better support their care recipients.

Given that some of the most common reasons for family caregivers not accessing their own patient portal included a preference for speaking directly with their provider and not having a patient portal or perceiving a need to use it, providers should also discuss the benefits and barriers to portal use among patients who identify as family caregivers and demonstrate its potential for web-based communication, which could be a fruitful alternative to direct face-to-face or telephone-based communication. These caregivers may benefit from information about the portal and its potential benefits for supporting them in managing their own health conditions and the needs of their relatives. More than one-fifth of caregivers reported that they did not perceive a need for the portal, highlighting the possibility that these caregivers, as patients themselves, may not see value in the patient portal or may not have health conditions that require the use of the portal. However, given the considerable research on caregiver distress associated with negative mental, physical, and psychosocial health outcomes, it may be valuable for providers to screen caregivers for distress or at least encourage their use of their own patient portal to keep track of their health and communicate with their providers about health concerns that may be related to caregiving responsibilities [30,31]. Doing so would also require discussions about the privacy and security of the portal, including potential uses of health information. Future research should continue soliciting insights from caregivers and patients about their concerns and preferences related to privacy and security of health information on the patient portal. For example, caregivers may view information that patients do not want them to know about; discussions involving clinicians, patients, and caregivers should be comprehensive in identifying preferences related to the amount and nature of information shared with caregivers and the instances during which information can be observed by caregivers.

Our finding that caregivers with depression were less likely to use their care recipient’s portal was in contrast with other recent research that finds the opposite relationship [32]. Although it is possible that the use of the care recipient’s patient portal is a way of gathering information if the depression is related to caregiving or that using the portal actually facilitates social engagement by enabling the caregiver to communicate with information or with the health care system, it is also possible that the use of a care recipient’s patient portal could actually exacerbate depression or anxiety—an area requiring further study.

### Limitations

There are some limitations to our study. First, HINTS is a cross-sectional survey that limits the determination of causality. Second, our sample was limited to the representation of rural respondents, which could bias our estimates of the relationship between geographic residence and caregiver use of the care recipient’s patient portal. This could be attributed to complexities in accessing mailing addresses for rural households to disseminate the survey and requires further study, as rural communities are particularly susceptible to disparities in digital access and may face additional barriers to accessing their care recipient’s patient portal [24]. Finally, the HINTS survey sample was limited, particularly in the representation of racial and ethnic minority groups. Racial and ethnic minority family caregivers may have different concerns about the patient portal or different reasons for using it. For instance, research suggests that Black and Hispanic caregivers, on average, spend more hours caregiving and are more likely to co-reside with their care recipient when compared with White, non-Hispanic caregivers [33]. As a result, these caregivers may not perceive the need to use their care recipient’s patient portal, may not have enough time to use their own, or may have different concerns about communicating via technology that need to be studied further.

### Conclusions

Our analysis suggests that family caregivers who use their own patient portals are more likely to use their care recipient’s patient portal. This suggests, first, that family caregivers may use the portal for older adults and other populations that have been previously described as having lower engagement with their patient portal. However, it also suggests that there is a need for policies and technology designs to facilitate family caregiver use of their own patient portal and encourage their use of their care recipient’s portal. Family caregivers’ engagement with their care recipient’s portal could be critical to achieving value from telehealth, supporting caregivers in their caregiving responsibilities, and supporting effective patient care.

## Appendix

Multimedia Appendix 1 Family caregivers’ technology ownership and engagement (n=294).

## Abbreviations

HINTS: Health Information National Trends Survey
OR: odds ratio
