# Supplementary material for: Evaluation of Family Caregivers’ Use of Their Adult Care Recipient’s Patient Portal From the 2019 Health Information National Trends Survey: Secondary Analysis
Source: JMIR Aging. 2021 Oct 4;4(4):e29074. doi: 10.2196/29074 (PMC8524330; doi:10.2196/29074)
Supplement: Multimedia Appendix 1 [file aging_v4i4e29074_app1.docx]

Family caregivers’ technology ownership and engagement (n=294)
